# Effect of Nutrient Enrichment on Alpha and Beta Diversity of Macroinvertebrate Community in a Boreal River of Northern China

**DOI:** 10.3390/biology15100816

**Published:** 2026-05-21

**Authors:** Xu Sun, Yuening Guo, Xiaochen Wang, Wenfei Li, Changhong Li, Yingbin Lou, Shanshan Cao, Zhongwei Wang, Zhenguo Li, Gang Liu

**Affiliations:** 1 Liaoning Provincial Key Laboratory for Hydrobiology, College of Fisheries and Life Science, Dalian Ocean University, Dalian 116023, China; sunxu61204@163.com (X.S.); 15937323607@163.com (Y.G.); wq18421@126.com (X.W.); 19531512861@163.com (W.L.); 2 Liaoning Dalian Ecology and Environment Monitoring Center, Dalian 116007, China; 15998493482@163.com (C.L.); jasonlou1102@163.com (Y.L.); 13555976553@163.com (S.C.); wanglaoer1980@hotmail.com (Z.W.); lzhg1999@163.com (Z.L.)

**Keywords:** nutrient enrichment, macroinvertebrates, functional homogenization, beta diversity, trait-based approach

## Abstract

Nutrient enrichment poses a major threat to freshwater ecosystems, yet integrated assessments of its effects on multiple dimensions of biodiversity remain limited. In this study, we investigated the responses of taxonomic, functional, and phylogenetic diversity (alpha and beta) of macroinvertebrate communities along a nutrient gradient in the Taizi River, a boreal river in northern China. The results revealed that macroinvertebrate abundance was 4.51 ind./m^2^, belonging to three phyla and 68 species. Total phosphorus (TP) was the primary environmental driver, reducing functional evenness and dispersion at the alpha level, thereby promoting functional homogenization. At the beta level, TP increased nestedness and decreased turnover, leading to the loss of endemic species and a community dominated by species with broad ecological tolerance. Structural equation modeling showed that TP exerted stronger effects on functional and phylogenetic diversity than on taxonomic diversity. These findings provide multidimensional insights into nutrient-driven biodiversity loss and suggest that TP should be prioritized in water quality management to prevent functional homogenization and maintain beta diversity turnover. Collectively, these findings inform targeted conservation strategies for nutrient-enriched river ecosystems.

## Simple Summary

Nutrient pollution is a serious problem in rivers worldwide. In this study, we investigated how extra nutrients, especially nitrogen and phosphorus, affect the tiny animals living at the bottom of the Taizi River in northern China. We found that too much phosphorus causes these animal communities to become more similar to each other, losing the uniqueness of the species that makes each part of the river special. Phosphorus also reduces the variety of roles these animals play in the ecosystem, making the river less healthy and resilient. In contrast, nitrogen had weaker effects. Our results show that controlling phosphorus pollution should be a top priority for protecting river life. By focusing on phosphorus, water managers can help preserve a wider range of species and the important jobs they do, which benefits water quality and the overall health of the river for people and nature.

## 1. Introduction

River ecosystems are characterized by high spatial and temporal heterogeneity, which supports diverse biological communities [1,2]. Nutrients concentration in water-bodies serve as key environment filters, influencing community structure through direct and indirect pathways. For example, phytoplankton respond rapidly to nutrient enrichment, often exhibiting increased biomass and dominance of a few taxa [3,4,5]. Such shifts alter physicochemical conditions [6,7] and exert bottom-up effects on higher trophic levels [8,9,10], ultimately driving biodiversity loss and biotic homogenization [11,12,13].

Macroinvertebrate assemblages have long been recognized as valuable bioindicators of aquatic ecosystem health, with sensitive taxa such as Ephemeroptera, Plecoptera, and Trichoptera (EPT) exhibiting rapid responses to changes in water quality [14,15,16]. Furthermore, their intermediate position in food webs enables them to link isolated water bodies spatially and serve as a crucial food source for higher trophic levels in both aquatic and adjacent riparian habitats [17,18]. Therefore, understanding how macroinvertebrate biodiversity responds to nutrient enrichment is essential for maintaining river ecosystem integrity.

Recent biodiversity research has emphasized the limitations of focusing on a single facet of diversity [19,20,21]. Integrative analyses of alpha and beta diversity provide complementary insights into community dynamics across spatial scales [22,23,24]. Moreover, decomposing beta diversity into turnover and nestedness components can reveal the underlying mechanisms structuring biodiversity patterns [25,26].

In addition to taxonomic diversity, functional and phylogenetic diversity offer complementary information by capturing species’ ecological roles and evolutionary distinctiveness, which are often overlooked by traditional taxonomic approaches [27,28,29]. Several studies have demonstrated that functional and phylogenetic metrics can provide greater sensitivity to environmental change [30,31,32], and partly complement information on taxonomic diversity [33,34].

Nitrogen (N) and phosphorus (P) often exert contrasting effects on aquatic communities due to their distinct roles in limiting primary production and their differential associations with anthropogenic sources [35,36,37]. In many temperate rivers, P is considered the primary limiting nutrient, whereas N enrichment may favor certain pollution-tolerant taxa [38,39,40]. While several studies have examined the effects of nutrient enrichment on individual facets of biodiversity, integrated assessments combining taxonomic, functional, and phylogenetic dimensions across both alpha and beta levels remain limited, particularly in the boreal rivers of northern China.

While recent studies have investigated the effects of nutrient enrichment on biodiversity, comprehensive analytical studies combining taxonomic, functional, and phylogenetic diversity at alpha and beta levels remain scarce. In addition, further research is warranted to explore the variation and interrelationships among these diversity dimensions across different spatial scales. To address this gap, we collected macroinvertebrate and environmental data from 41 sites along the Taizi River—a typical watershed with a wide trophic gradient in northern China. We tested three hypotheses: (1) total phosphorus (TP) exerts a stronger influence on macroinvertebrate community structure than total nitrogen (TN); (2) increasing TP concentrations drive functional homogenization of macroinvertebrate assemblages; and (3) TP has a more pronounced effect on functional and phylogenetic diversity than on taxonomic diversity.

## 2. Materials and Methods

### 2.1. Study Area

**Figure 1:**
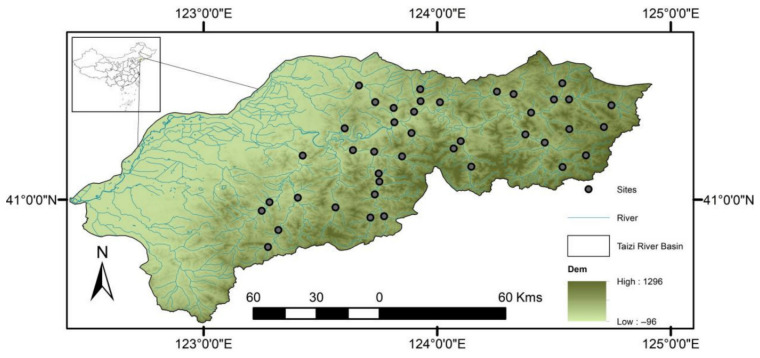
Distribution of sampling points in the study area. Map was created using ArcGIS software by Esri (Environmental Systems Resource Institute, ArcGIS 10.8; https://www.esri.com/en-us/home (accessed on 15 May 2018).

Taizi River is located in Northeast China, with an east–west orientation. Its geographical location is 122°26′–124°53′ E and 40°29′–41°39′ N, with a total length of 413 km and average depth 0.24 m (Figure 1). Its drainage area is 1.39 × 10^4^ m^2^, with a flow rate of 106 m^3^/s. Roughly 70% of the drainage area is mountainous, with a vegetation coverage of 70% and a forest coverage of approximately 50%. The basin is located in the temperate semi-humid monsoon climate zone, characterized by alternating cold and warm temperatures, as well as dry and wet seasons. With the rapid socioeconomic development of the region, the concentrations of nutrients in the basin have significantly increased, which directly affects aquatic biodiversity [41]. While concerns regarding the ecological health of the watershed have been raised, the impact of nutrient enrichment on aquatic biodiversity in the watershed remains elusive [42].

### 2.2. Field Sampling

In April 2018, macroinvertebrate samples were collected from 41 sites across the Taizi River Basin. At each site, five replicate samples were taken within a 100 m reach using a Surber net (0.09 m^2^, 500 µm mesh, Beijing Haifuda Technology Co., Ltd., Beijing, China), covering different habitat types (e.g., riffles, pools, and runs). Samples were washed through a 500 µm sieve, sorted manually in white enamel trays, and preserved in 70% ethanol. In the laboratory, specimens were identified to the genus or species level using standard taxonomic keys [43,44,45,46]. Water samples were collected at a depth of 20 cm below the surface and analyzed for total nitrogen (TN), total phosphorus (TP), ammonia-N (NH_4_^+^-N), dissolved oxygen (DO), pH, conductivity (COND), chemical oxygen demand (COD_Mn_), and water temperature (WT) following standard methods [47].

### 2.3. Data Analyses

#### 2.3.1. Alpha and Beta Diversity

Taxonomic α-diversity was quantified using species richness (SR), Shannon index (H), Simpson index (GS), and evenness (J) via the ‘vegan’ package in R. Functional α-diversity indices—functional richness (FRic), functional evenness (FEve), functional divergence (FDiv), functional dispersion (FDis), and Rao’s quadratic entropy (RaoQ)—were calculated using the ‘FD’ package, based on nine species traits (Table 1). Phylogenetic α-diversity was estimated as Faith’s phylogenetic diversity (PD) using the ‘picante’ package, based on a phylogenetic tree constructed from the macroinvertebrate taxonomic backbone. Likewise, taxonomic, functional, and phylogenetic β-diversity were each partitioned into total β-diversity, turnover, and nestedness components using the ‘beta’ function in the ‘BAT’ package. Macroinvertebrate functional groups are divided in Table 1.

#### 2.3.2. Statistical Analyses

To reflect the degree of eutrophication and organic pollution of the water body, as well as to infer external pollution inputs and self-purification capacity, the environmental physical and chemical factors included the following: total phosphorus (TP), total nitrogen (TN), ammonia nitrogen (NH_4_^+^-N), dissolved oxygen (DO), pH value (pH), conductivity (COND), potassium permanganate (COD_Mn_), and water temperature (WT) (Table 2).

Principal component analysis (PCA) was performed to identify key environmental variables structuring macroinvertebrate communities. Environmental variables were log-transformed where necessary to meet normality assumptions. The relationships between nutrients (TN, TP) and diversity indices were assessed using Mantel tests and linear regression models. Variation partitioning (VPA) was conducted using the ‘varpart’ function to quantify the unique and shared contributions of TN and TP to diversity variation.

To characterize the biodiversity patterns and their responses to environmental gradients, multiple ecological indices were calculated across taxonomic, functional, and phylogenetic dimensions, including both α-diversity (within-community diversity) and β-diversity (among-community diversity) metrics (Table 3).

Structural equation modeling (SEM) was constructed using the “lavaan” package to test the direct and indirect effects of TN and TP on taxonomic, functional, and phylogenetic diversity. Model fit was evaluated using χ^2^/df, comparative fit index (CFI), and root mean square error of approximation (RMSEA). Acceptable fit was defined as χ^2^/df < 3, CFI > 0.90, and RMSEA < 0.08. All analyses were performed in R version 4.3.

## 3. Results

### 3.1. Macroinvertebrate Community Composition

Macroinvertebrates species were collected with average abundance 4.51 ind./m^2^, belonging to three phyla and 68 species (Table 4), among which Arthropoda comprised the highest number of species (53 species), followed by Mollusca (10 species) and Annelida (5 species).

### 3.2. Analysis of Environmental, Physical, and Chemical Factors

**Figure 2:**
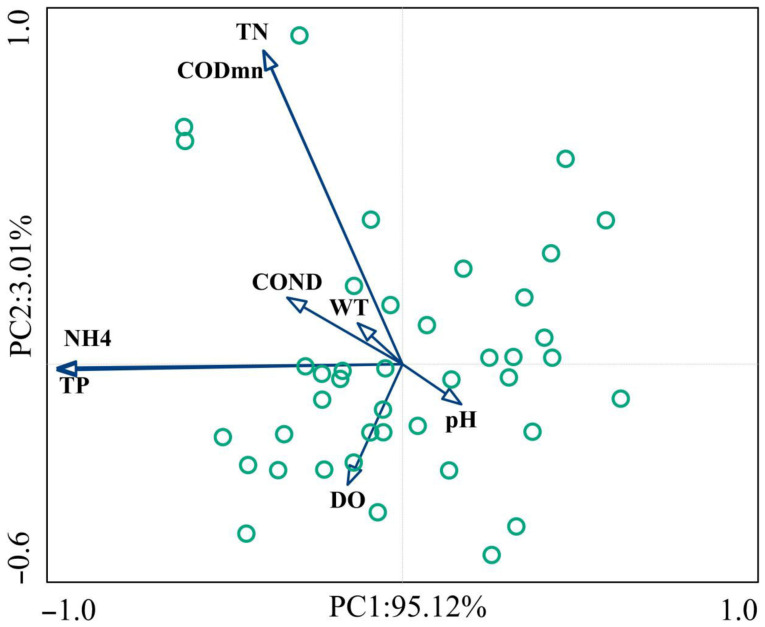
Principal component analysis results. Physical and chemical factors of water: TP, TN, ammonia nitrogen (NH_4_^+^-N), dissolved oxygen (DO), pH, conductivity (COND), potassium permanganate (COD_Mn_), and water temperature (WT). Note: the circle means sampling sites, the arrow means physical and chemical fators.

Principal component analysis (PCA) was performed to identify key environmental factors influencing the composition and structure of macrobenthic invertebrates using eight physical and chemical factors, namely total phosphorus content (TP) content, total nitrogen content (TN), ammonia nitrogen content (NH_4_^+^-N), dissolved oxygen (DO), pH, conductivity (Cond), chemical oxygen demand (COD_Mn_), and water temperature (WT). The results revealed that the first two principal components explained 92.07% of the total variance (PC1: 70.52%, PC2: 21.55%). TP, TN, and NH_4_^+^-N were the primary contributors to PC1, while DO and WT contributed most to PC2 (Figure 2).

**Figure 3:**
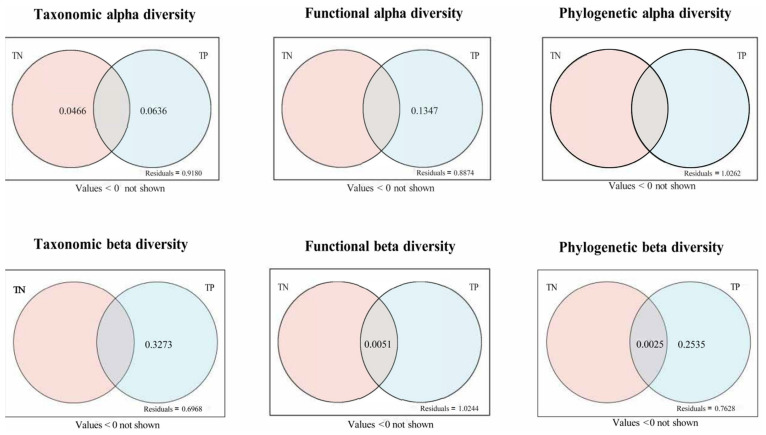
Decomposition analysis of variance of total nitrogen and total phosphorus on taxonomic, functional, and phylogenetic diversity of macroinvertebrates. Values less than 0 will not be displayed.

According to the results of variance partitioning analysis, concerning alpha diversity, TN accounted for 6.57% of taxonomic variation and 5.76% of functional variation, whilst TP accounted for 0.69% and 8.46% of taxonomic and functional variation, respectively. The combined explanatory power of TN and TP for taxonomic, functional, and phylogenetic variation was 2.14%, 3.56%, and 0.49%, respectively (residuals = 0.9180, 0.8874, 1.0262). Moreover, regarding beta diversity, TP accounted for 30.73%, 0.81%, and 27.66% of taxonomic, functional, and phylogenetic variation, respectively. The combined explanatory power of TN and TP for taxonomic variation was 1.83%, with residuals of 0.6968, 1.0244, and 0.7628, respectively (Figure 3).

### 3.3. Relationship Between Biodiversity and Environmental Factors

**Figure 4:**
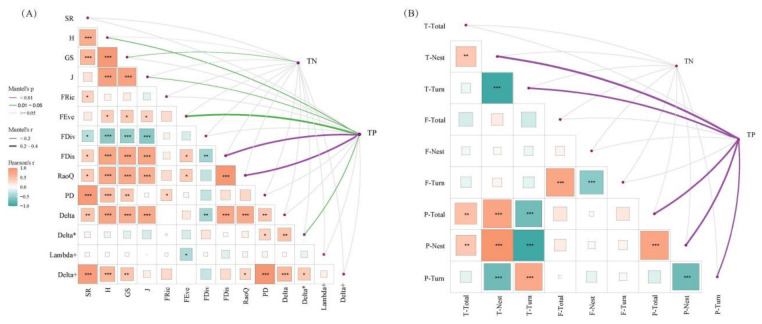
Mantel analysis of total nitrogen and total phosphorus on the alpha, beta diversity of macroinvertebrates. (**A**) Alpha diversity. (**B**) Beta diversity. Note: Delta: taxonomic diversity, Delta*: taxonomic distinctness, Lambda+: variation in taxonomic distinctness, Delta+: weighted taxonomic diversity. Note: *** *p* < 0.001, ** *p* < 0.01, * *p* < 0.05.

**Figure 5:**
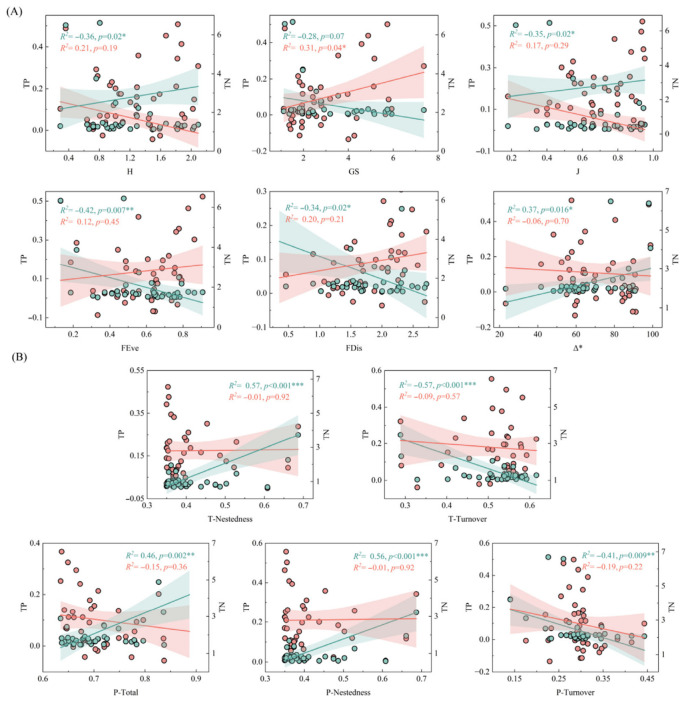
Linear regression model of total nitrogen and total phosphorus with taxonomic, functional, and phylogenetic diversity of macroinvertebrates. (**A**): Alpha diversity; (**B**): beta diversity; red and green represent the trends of total nitrogen and total phosphorus, respectively. Note: *** *p* < 0.001, ** *p* < 0.01, * *p* < 0.05.

Mantel tests and linear regression analyses revealed that TP was significantly negatively correlated with functional evenness (FEve, *p* = 0.007) and functional dispersion (FDis, *p* = 0.02), supporting the hypothesis of TP-induced functional homogenization (Figure 4A and Figure 5A). In contrast, TN was positively correlated with Shannon index (*p* = 0.005) and evenness (*p* = 0.004), suggesting a shift toward pollution-tolerant taxa under high TN conditions (Figure 5A). At the β-diversity level, TP was significantly positively correlated with taxonomic and phylogenetic nestedness (*p* < 0.001) and negatively correlated with turnover components (*p* < 0.001), indicating a shift from species replacement to species loss along the TP gradient (Figure 4B and Figure 5B). TN showed no significant correlation with β-diversity components (*p* > 0.05).

### 3.4. Impact of Nutrients on Macroinvertebrate

**Figure 6:**
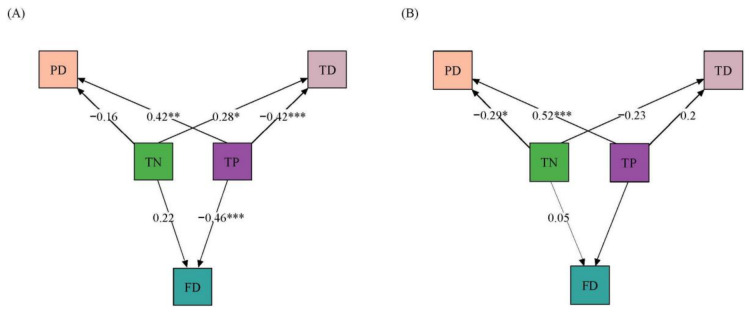
SEM analysis of macroinvertebrates based on total nitrogen and total phosphorus. (**A**): Alpha diversity; (**B**): beta diversity. Note: *** *p* < 0.001, ** *p* < 0.01, * *p* < 0.05.

The results of SEM (Figure 6) unveiled that nutrients affected different aspects of macroinvertebrate diversity in various ways. At the alpha diversity level, TN significantly positively affected TD (R = 0.28, *p* < 0.05 *), while TP exerted a highly significant positive effect on PD (R = 0.42, *p* < 0.01 **) and a highly significant negative effect on TD and FD (R = −0.42, *p* < 0.001 ***; R = −0.46, *p* < 0.001 ***). At the beta diversity level, TN significantly negatively affected PD (R= −0.29, *p* < 0.05 *), whereas TP demonstrated an extremely significant positive effect on PD (R = 0.52, *p* < 0.001 ***).

## 4. Discussion

This study provides a comprehensive assessment of how nutrient enrichment (using two factors: total nitrogen and total phosphorus content) affects multiple dimensions of macroinvertebrates diversity in the Taizi River Basin. Our results demonstrate that TP is the dominant driver of community change, leading to functional homogenization at the α-diversity level and increased nestedness at the β-diversity level. These findings align with the growing recognition that phosphorus, rather than nitrogen, often limits primary production in freshwater ecosystems, and its enrichment can trigger cascading effects on higher trophic levels [5]. Specifically, PCA and variance decomposition analysis identified TP and TN as the primary trophic factors affecting macroinvertebrate diversity. Overall, total phosphorus largely accounted for the variation in macroinvertebrate diversity at the alpha and beta levels, while the interaction between total nitrogen and total phosphorus accounted for some of the variation in phylogenetic beta diversity. Our study indicated that as the most important environmental factor, the increase in total phosphorus level might aggravate the homogenization of the benthic community in this area, hallmarked by a significant decrease in the functional diversity index (FEve, FDis) of the community. Moreover, the nested component was enhanced at the beta diversity level, accompanied by a reduced turnover component. Although the community structure was generally stable, this stability may come at the expense of species diversity. Indeed, simplification of species composition may reduce community resistance to environmental changes [48,49].

Given the large spatial variation in total phosphorus and total nitrogen concentrations in the study area, we hypothesized that macroinvertebrate diversity decreased with nutrient enrichment, potentially leading to functional homogenization [50,51]. Herein, the results of regression analysis demonstrated that elevated total nitrogen levels increased the taxonomic diversity of macroinvertebrates, especially for those species that could use additional nitrogen sources [52]. For example, the Shaker family, the most abundant taxon in the study area, was followed by the Trematode family, which comprises predominantly nitrogen-loving species [53]. The increase in taxonomic diversity with TN likely reflects the proliferation of pollution-tolerant taxa (e.g., Chironomidae and Tubificidae), which may artificially inflate species richness without enhancing functional diversity. However, this increase may not be evenly distributed across all species. An increase in species richness does not reflect an increase in functional diversity, given that functional diversity is largely determined by the distribution and abundance of functional traits, which was also validated in subsequent analyses [54,55].

The observed decline in functional evenness (FEve) and functional dispersion (FDis) with increasing TP suggests that only a subset of functional traits persists under high nutrient conditions. This functional homogenization is consistent with previous studies in eutrophic systems and implies a loss of ecological redundancy, potentially reducing community resilience to future disturbances [7]. Notably, total phosphorus levels exerted a more significant influence on functional diversity and beta diversity. According to the results of regression analysis, increasing total phosphorus concentrations were correlated with decreased macroinvertebrate functional evenness and turnover components and increased nested components of taxonomic and phylogenetic beta diversity.

At the β-diversity level, the shift from turnover to nestedness along the TP gradient indicates that sites with high phosphorus levels support only a subset of the species pool, predominantly generalist taxa. This pattern is typical of environmental filtering, where harsh conditions exclude sensitive species, leading to biotic homogenization across sites [8,12]. Furthermore, the absence of a significant correlation between TN and β-diversity further underscores the primary role of TP in shaping regional community structure in this system. In this study, the analysis of Mantel’s results uncovered a strong correlation between taxonomic and phylogenetic beta diversity [56], implying the emergence of specific phylogenetic patterns as nutrients are enriched within the community [57]. Nevertheless, the simultaneous increase in species’ taxonomic and phylogenetic diversity does not necessarily correspond to an increase in the number of species performing different functions [58,59]. At the same time, the decline in functional evenness corroborated the rapid dominance of certain functional traits in the community, such as the increase in direct collectors and the increase in the number of collectors, likely ascribed to the accumulation of organic matter such as plankton or detritus caused by slow regional flow rates [18,60]. Consequently, species performing specific functions appear to have been lost or replaced [61,62], resulting in functional redundancy. This homogenization may compromise the stability of communities and their ability to cope with environmental changes, potentially affecting the level of ecosystem functioning [7,63].

**Figure 7:**
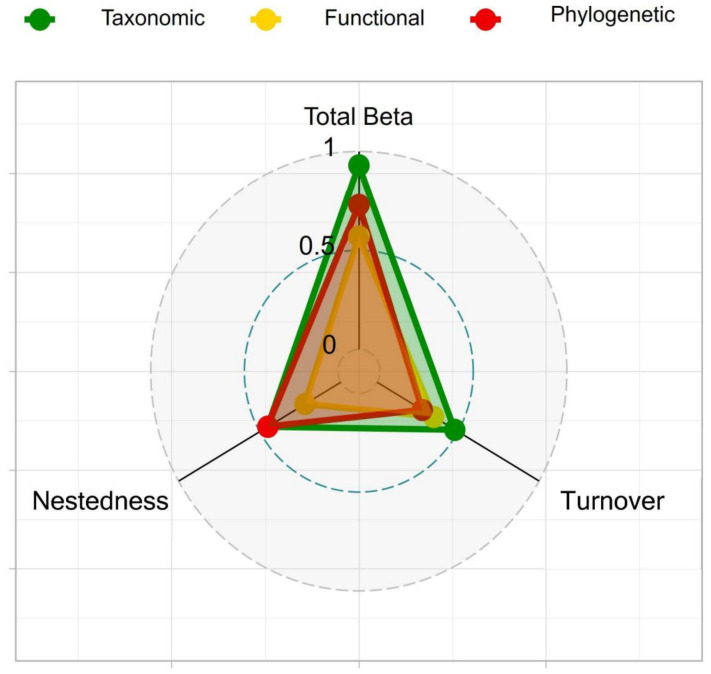
Macroinvertebrate total beta diversity and its turnover, nestedness components.

More importantly, the radar map illustrated that the taxonomic beta diversity was the highest (0.93), followed by the phylogenetic beta diversity (0.73) and the functional beta diversity (0.57) (Figure 7). The proximity of taxonomic diversity was close to 1, suggesting that each site harbored a unique species composition when species abundance was not considered. Interestingly, the turnover component of phylogenetic beta diversity was lower than that of functional beta diversity, indicating that the evolutionary convergence of species was weaker than their functional convergence [64]. While these species may originate from different evolutionary branches, they exhibit convergence in functional traits as a result of adaptation to similar environments [65]. Conversely, the turnover component of functional beta diversity was more dominant than the nested component. In other words, paired species substitutions led to a shift in most functional traits, resulting in differences in functional beta [66]. Overall, the variability noted across different aspects of beta diversity was consistent with the findings of previous studies and emphasizes the importance of considering functional and phylogenetic in beta diversity research [25,27,67].

In line with our third hypothesis, elevated total phosphorus levels exhibited a significantly stronger influence on the functional and phylogenetic diversity of macroinvertebrates compared to taxonomic diversity [37,68]. This pattern is supported by our analytical findings, as evidenced by the data presented in Figure 6. From the perspective of the structural equation model, there was a more significant correlation between functional diversity and nutrients, followed by taxonomy and phylogenetic diversity. This further reinforces the hypothesis that environmental factors filter traits rather than species [67,69]. However, the results were reversed upon considering when beta diversity. The correlations between taxonomic and phylogenetic beta diversity and nutrients were high, whereas those between functional beta diversity and nutrients were low and non-significant. At the same time, the results of Mantel analysis demonstrated a high correlation in the same aspect of diversity, such as between GS and H, whereas the correlation between different aspects of diversity was low, signaling that they all individually characterize the unique information of diversity [70]. Thus, accounting for functional and phylogenetic diversity can provide complementary information beyond taxonomic diversity, such as evolutionary relationships between ecosystem functions and species [27]. Of note, alpha diversity does not account for differences in species composition between sites, making direct comparisons challenging. In contrast, beta diversity accurately addresses this shortcoming by identifying differences between sites, thereby effectively capturing temporal and spatial differences in community responses [71,72]. Based on the different results revealed by different diversity indexes, we speculate that considering multiple dimensions of diversity can expand our understanding of community dynamics [73,74]. Overall, this study explored the role of environmental filtering driven by nutrient enrichment, and future long-term studies in the region are warranted to validate our results and provide insights for environmental management.

TP serves as a bulk proxy for the overall nutrient pool, and its correlation with biological responses in our study likely reflects the strong covariance between TP and bioavailable phosphorus fractions under the local hydrological and biogeochemical conditions. This distinction is important because the use of TP as a causal indicator can sometimes obscure the underlying mechanisms of nutrient–biota interactions [75]. Large-scale trait databases have facilitated the widespread application of functional diversity metrics in freshwater bioassessment, and our study benefited from such trait-based approaches to detect community-level functional responses along the TP gradient [76]. We caution that TP itself does not directly influence phytoplankton or macroinvertebrates; rather, it serves as a composite metric of trophic state [77]. The actual bioavailable phosphorus fraction (dissolved reactive phosphorus) is the key driver of algal production, but its measurement was not available in our dataset. Hence, our interpretations of TP effects should be understood as reflecting the overall nutrient enrichment gradient, within which the dissolved phosphorus fraction covaries strongly with TP. In addition, our findings further reveal that the apparent effect of total phosphorus on macroinvertebrate functional diversity is primarily indirect, mediated by reduced dissolved oxygen. Low dissolved oxygen directly stresses aquatic macroinvertebrates, particularly taxa with high respiratory demands (EPT), and favors tolerant taxa with specific functional traits. The loss of oxygen-sensitive species reduces functional evenness and dispersion, as the community becomes dominated by a few tolerant groups that occupy a narrower range of functional niches—a process of functional homogenization. Thus, while TP serves as a useful integrative indicator of nutrient pressure, it is the cascading effect on oxygen availability that more directly drives the observed biodiversity patterns. Future studies should incorporate high-frequency DO measurements alongside nutrient data to better disentangle these interacting drivers.

## 5. Conclusions

This study investigated the effects of nutrients enrichment on macroinvertebrate diversity across taxonomic, functional, and phylogenetic dimensions in a boreal river of northern China. Our results demonstrate that TP is the primary driver of biodiversity change, promoting functional homogenization at the alpha level and increasing nestedness at the beta level. Functional and phylogenetic diversity were more sensitive to nutrient enrichment than taxonomic diversity, underscoring the importance of incorporating multiple diversity facets in ecological assessments.

From a management perspective for Taizi River Basin,, we recommend that firstly, given the dominant role of TP in driving functional homogenization and nestedness, phosphorus control should be prioritized over nitrogen management to preserve macroinvertebrate biodiversity. Secondly, we recommend the protection of high-turnover and low TP levels sites, as these sites contribute disproportionately to regional β-diversity and may serve as refugia for sensitive species. Finally, future monitoring efforts should incorporate functional and phylogenetic metrics alongside traditional taxonomic indices to provide a more comprehensive assessment of ecosystem health. These strategies can help safeguard macroinvertebrate diversity and ecosystem function in nutrient-impacted river systems. In the future, studies should include direct measurements of DO dynamics along the eutrophication gradient to better resolve causal pathways.

## Figures and Tables

**Table 1:** Functional groups of macroinvertebrate divided by nine species traits [26].

Trait Group	Trait	Trait State (Modality)	Code
Habi	Habit	Burrow	Habi1
		Climb	Habi2
		Sprawl	Habi3
		Cling	Habi4
		Swim	Habi5
		Skate	Habi6
Resp	Respiration	Tegument	Resp1
		Gills	Resp2
		Plastron, spiracle (aerial)	Resp3
Drft	Occurrence in drift	Rare (catastrophic only)	Drft1
		Common (typically observed)	Drft2
		Abundant (dominant in drift samples)	Drft3
Rheo	Rheophily	Depositional only	Rheo1
		Depositional and erosional	Rheo2
		Erosional	Rheo3
Ther	Thermal preference	Cold stenothermal or cool eurythermal	Ther1
		Cool/warm eurythermal	Ther2
Trop	Trophic habit	Collector-gatherer	Trop1
		Collector-filterer	Trop2
		Herbivore (scraper, piercer, and shedder)	Trop3
		Predator (piercer and engulfer)	Trop4
		Shredder (detritivore)	Trop5
Disp	Female dispersal	Low (<1 km flight before laying eggs)	Disp1
		High(>1 km flight before laying eggs)	Disp2
Size	Size at maturity	Small (<9 mm)	Size1
		Medium (9–16 mm)	Size2
		Large (>16 mm)	Size3
Vlot	Voltinism	Semivoltine (<1 generation/y)	Vlot1
		Univoltine (1 generation/y)	Vlot2
		Bi- or multivoltine (>1 generation/y)	Vlot3

**Table 2:** Environmental physical and chemical factors in Taizi River.

Factors	Abbreviation	Description
Total phosphorus	TP	Eutrophication of water bodies
Total nitrogen	TN	
Ammonia nitrogen	NH_4_^+^-N	
Dissolved oxygen	DO	Organic pollution consumes oxygen
Potassium permanganate	COD_Mn_	
pH value	pH	Pollution source and process indication
Conductivity	COND	
Water temperature	WT	

**Table 3:** Alpha and beta diversity index across taxonomic, functional, and phylogenetic dimensions.

	Index	Abbreviation	Description
Taxonomic alpha diversity	Species Richness	SR	Total number of species within a community
	Shannon–Wiener index	H′	Reflects species richness and evenness; sensitive to rare species
	Simpson Index	GS	Reflects community dominance and diversity; sensitive to dominant species
	Pielou Index	J	Measures the distribution evenness of individual abundance among species
Functional alpha diversity	Functional Richness	FRic	The magnitude of functional trait space occupied by species in the community
	Functional Evenness	FEve	Distribution evenness of functional traits in trait space
	Functional Divergence	FDiv	Differentiation and dispersion degree of functional traits among community species
	Functional Dispersion	FDis	Average dispersion of species away from the community centroid in trait space
	Rao’s Quadratic Entropy	RaoQ	Integrates species abundance and trait dissimilarity to quantify functional diversity
Phylogenetic alpha diversity	Phylogenetic Diversity	PD	Total branch length of phylogenetic lineages; reflects evolutionary historical diversity
Taxonomic beta diversity	Total Taxonomic β-diversity	T-Total	Overall compositional dissimilarity among communities
	Taxonomic Nestedness	T-Nest	Compositional difference derived from species loss/gain (nestedness process)
	Taxonomic Turnover	T-Turn	Compositional difference derived from species replacement (turnover process)
Functional beta diversity	Total Functional β-diversity	F-Total	Overall functional trait dissimilarity among communities
	Functional Nestedness	F-Nest	Functional difference derived from trait loss/gain (nestedness process)
	Functional Turnover	F-Turn	Functional difference derived from trait replacement (turnover process)
Phylogenetic beta diversity	Total Phylogenetic β-diversity	P-Total	Overall phylogenetic compositional dissimilarity among communities
	Phylogenetic Nestedness	P-Nest	Phylogenetic difference derived from lineage loss/gain (nestedness process)
	Phylogenetic Turnover	P-Turn	Phylogenetic difference derived from lineage replacement (turnover process)

**Table 4:** List of macroinvertebrates in Taizi River.

Phyla	Species	Abundance (ind./m^2^)
Arthropoda	*Ephemera orientalis*	37.60
	*Hepyageniidea* sp.	4.13
	*Baetis* sp.	9.20
	*Epeorus herklotsi*	2.53
	*Cinygmina yixingensis*	10.93
	*Leptophlebiidae* sp.	2.00
	*Caenis nigropunctata*	2.27
	*Siphlonurus* sp.	0.67
	*Drunella* sp.	0.53
	*Serratella rufa*	0.40
	*Prosopistoma sinense*	0.13
	*Perla* sp.	0.67
	*Nemoura* sp.	3.47
	*Hydropsyche* sp.	6.80
	*Stenopsychida* sp.	1.33
	*Parakiefferiella torutata*	4.00
	*Procladius choreus*	1.07
	*Eukiefferiella brehmi*	10.13
	*Cricotopus albiforceps*	0.80
	*Paracricotopus tamabrevis*	2.13
	*Parametrionemus stylatus*	4.40
	*Cricotopus annulator*	12.13
	*Cricotopus bicinctus*	2.67
	*Sympotthastia takatensis*	1.60
	*Orthocladius wetterensis*	8.67
	*Orthocladius mixtus*	12.80
	*Rheocricotopus fuscipes*	1.47
	*Hydrobaenus kondoi*	8.53
	*Paracladius conversus*	1.87
	*Diamesa insignipes*	0.27
	*Cricotopus vierriensis*	0.67
	*Potthastia montium*	9.87
	*Tipula* sp.	1.60
	*Tabanus* sp.	2.80
	*Ceratopogonidae* sp.	0.40
	*Psychoda* sp.	1.07
	*Simulidae* sp.	0.40
	*Tipulidae* sp.	0.27
	*Eubrianax* sp.	0.13
	*Cybister* sp.	0.13
	*Ischnura asiatica*	0.13
	*Lestes* sp.	0.53
	*Macromia clio*	0.40
	*Aeschna sieboldii*	0.40
	*Aeschna* sp.	3.33
	*Sympetrum infuscotum*	0.67
	*Aphelochirus* sp.	0.40
	*Naucoridae* sp.	15.60
	*Notonecta* sp.	0.13
	*E.modestus*	5.60
	*Palaemonetes sinensis*	7.87
	*Mysis* sp.	17.07
	*Gammarus*	17.47
Mollusca	*Radix auricularia*	2.67
	*Radix tagotis*	3.20
	*Radix clessini*	1.20
	*Radix ovata*	0.27
	*Hippeutis umibilicalis*	1.60
	*Bellamya purificata*	0.13
	*Assimineidae* sp.	0.13
	*Bithynia* sp.	0.67
	*Corbicula fluminea*	0.27
	*Anodonta arcaeformis*	0.13
*Annelida*	*Limnodrilus hoffmeisteri*	24.53
	*Limnodrilus claparedianus*	21.73
	*Whitmania pigra*	0.40
	*Barbronia weberi*	3.33
	*Glossiphonia complanata*	4.27
Average		4.51

## Data Availability

Data will be made available upon request.
